# Supplementary material for: How to choose the size of facial areas of interest in interactive eye tracking
Source: PLoS One. 2022 Feb 4;17(2):e0263594. doi: 10.1371/journal.pone.0263594 (PMC8815978; doi:10.1371/journal.pone.0263594)
Supplement: Attachment — Submitted filename: r0_2021.docx [file pone.0263594.s001.docx]

In this study, the authors simulate fixations on faces with different accuracies (noise levels of the simulated Eye-Tracker). They then analyze how well fixation location in different ROIs can be estimated using four ROIs and three ROI-sizes. They quantify both type-1 and type-2 errors. They find the expected patterns that high accuracy is good, large ROIs lead to higher type-2 errors, but lower type-1 errors, but importantly, they give a clear framework and open-source tool on how to quantify these intuitions. Consequently, this helps in planning and intuiting eye-tracking face-perception studies with eye-trackers of different accuracy.
As the authors state in the paper, a limitation is, that the ROI-size, eye-tracking accuracy and stimulus sizes are all connected via viewing distance, and one can be traded off each other’s. A limitation in their type-2 error analysis is, that it hinges heavily on the placement of the “wrong” fixation point (in this case the forehead). But this is clearly visible from the paper. I encourage the authors to polish their tool a bit, because I think it could be very useful for the community.

I enjoyed reading this paper and I have only some minor comments.

**Strengths**

- **Clear hypotheses**
- **Solid simulation**
- **Simulation tools provided**

**Weaknesses**

- Code not available

Below you can find all of my comments.

Signed, Benedikt Ehinger

**Limitations of my review**

- **I am no expert in AOI or Face-Studies**

**Minor**

- Unclear definition of “fixation point”.
  As I understand from the data, you check for each simulated sample, whether it is inside or outside the AOI. I understood fixation point more as the average x/y of a fixation. “Gaze sample” could be a better term?
- Related to the above: Often, accuracies of eye trackers are calculated on fixation level and not on sample, thus counteracting potential influences of bad precision. It did not become clear to me, whether we should count the 120 samples as 120 Fixations, or as a single fixation.
- Thanks to the shared data, I reproduced some part of the main analyses of the paper

This replicates Figure 3b, but in reverse order of the x-axis and not as stacked barplots. Note that the means perfectly match Figure 3b – I additionally depicted the variability over subjects, which I think show the relation to Figure 1a, the gamma distributions of “base” accuracy”. Maybe this variability is of interest to the readers as well, quite often we aggregate too much and do not show the variability; but in this case I’m actually not sure.

Julia code: https://gist.github.com/behinger/e48029ca3d514869905b68275a74468f

- It would be super valuable, if you could host the source code of the simulation tool on github (or other platform + zenodo link for archiving), so that other researchers can build upon your work. Further, there is no documentation on how to use the tool (or did I miss it?), and I couldn’t get it to run properly. If documentation is too time-consuming, a short screencap would already help.
- On a similar note, it would be nice to get a short readme file in your data.zip, simply depicting what column means what. I think I figured it out, but I was confused at first.
- Plotting Suggesting: Maybe you can add the sizes of the stimuli (2.8°/1.9°) to one (or all?) stimulus depictions? That would help in setting the 1.5° accuracy better into relation.
- L319/L320: repetition of “essential”
- L113 was => were
